# Associations between testicular hormones at adolescence and attendance at chlorinated swimming pools during childhood

**DOI:** 10.1111/j.1365-2605.2011.01174.x

**Published:** 2011-10

**Authors:** M Nickmilder, A Bernard

**Affiliations:** Laboratory of Toxicology and Applied Pharmacology, Faculty of Medicine, Catholic University of LouvainBrussels, Belgium

**Keywords:** chlorination, chlorine, reproductive health, swimming pool, testes

## Abstract

The goal was to evaluate the associations between testicular hormones at adolescence and the exposure to chlorination by-products when attending chlorinated swimming pools. We obtained serum samples from 361 school male adolescents (aged 14–18 years) who had visited swimming pools disinfected with chlorine or by copper–silver ionization. We analysed serum concentrations of inhibin B (two different assays), total and free testosterone, sex hormone-binding globulin, luteinizing hormone (LH), follicle stimulating hormone (FSH) and dehydroepiandrosterone sulphate (DHEAS). There were strong inverse associations between serum levels of inhibin B (both assays) or of total testosterone, adjusted or unadjusted for gonadotropins and the time adolescents had spent in indoor chlorinated pools, especially during their childhood. Adolescents having attended indoor chlorinated pools for more than 250 h before the age of 10 years or for more than 125 h before the age of 7 years were about three times more likely to have an abnormally low serum inhibin B and/or total testosterone (<10th percentile) than their peers who never visited this type of pool during their childhood (odds ratio, 95% CI, 2.83, 1.06–7.52, *p* = 0.04 and 3.67, 1.45–9.34, *p* = 0.006, respectively). Such associations were not seen with free testosterone, LH, FSH and DHEAS or with the attendance of outdoor chlorinated pools or of the copper–silver pool. Swimming in indoor chlorinated pools during childhood is strongly associated with lower levels of serum inhibin B and total testosterone. The absorption of reprotoxic chlorination by-products across the highly permeable scrotum might explain these associations.

## Introduction

Disinfection of drinking or recreational water with chlorine results in the formation of a wide range of potentially toxic by-products among which the most abundant are the chloramines and various short-chain halogenated hydrocarbons such as trihalomethanes (THMs), haloacetic acids (HAAs) and haloacetonitriles (HANs) (Bull, 2000; World Health Organization, 2000; Weaver *et al.*, 2009). These chlorination by-products (CBPs) arise when active chlorine (hypochlorite/hypochlorous acid) destroys organic matter brought to water by natural or anthropogenic sources. Halogenated hydrocarbons derive mainly from the chlorination of humic acids and other natural organic compounds commonly found in drinking or recreational water. The chloramines, by contrast, typically form in swimming pools, especially in public pools, because of the reaction of chlorine with urea and other nitrogenous substances from the urine, sweat or saliva of bathers.

Rodent studies have demonstrated that at high doses several CBPs among the HAAs, HANs and THMs can damage the testes and disrupt spermatogenesis (Smith *et al.*, 1986; Linder *et al.*, 1994, 1995, 1997; Graves *et al.*, 2001; Christian *et al.*, 2002; Abdel-Nahab, 2003). Although there is a widespread human exposure to these potential reprotoxicants, to date, only two epidemiological studies have assessed their possible impact on the testicular function. Investigating a small cohort of healthy volunteers, Fenster *et al.* (2003) found that exposure to THMs in tap water was associated with decreased sperm mobility but these findings were not confirmed by Luben *et al.* (2007). These two studies, however, like most investigations in animals, addressed mainly the risks via the consumption of drinking water, a source of exposure, which might not be the most relevant for the male reproductive system. The reprotoxic risks of CBPs, if any, should rather be sought among subjects regularly attending swimming pools disinfected with chlorine. Because of their high level of organic pollution, chlorinated swimming pools contain indeed much higher levels of CBPs than tap water. In addition, when swimming, the pattern of human exposure shifts from ingestion to skin absorption, which for most lipophilic CBPs such as chloroform can account for more than 80% of the total uptake (World Health Organization, 2000). The dermal route exposure to CBPs might be particularly critical for the male reproductive system given the remarkable permeability of the scrotal skin (Fisher, 1989).

The objective of this study was to explore the relationships between various serum reproductive biomarkers and the exposure to CBPs when attending chlorinated pools. Our study based on school adolescents focused on the exposure during childhood when the male reproductive system is most vulnerable to hormone dysregulation. The existence in Belgium of a copper–silver pool in activity for more than 20 years enabled us to identify a reference population of swimmers with no or a minimal exposure to CBPs.

## Methods

### Population

The study participants were students who were examined in a cross-sectional study designed to assess the health impact of environmental pollution. The study used a wide range of sensitive biomarkers or tests to detect effects of environmental stressors (e.g. heavy metals, chlorination products, combustion products, allergens, etc.) on the respiratory tract, the immune system, the kidney and the testes. A total of 361 boys aged 14–17 years were examined in three secondary schools in Belgium located in the cities of Louvain-la-Neuve, Lessines and Bastogne. The participation rate was on average 72.2% and did not vary much between the schools (67.6, 74.5 and 77.5% respectively). The protocol of the study was described in detail elsewhere (Bernard *et al.*, 2008; Sixth European Union Framework Programme for Research). Briefly, parents completed a questionnaire inquiring about the health of their child and all known or suspected lifestyle or environmental factors likely to affect the reproductive system. The questionnaire comprised questions intended to estimate the total time the child had spent in indoor or outdoor chlorinated pools disinfected with chlorine or another method. The examination of students, performed in schools between 9:00am and 4:00 pm, included the measurement of height and body weight and the collection of a blood sample. The time of blood sampling was noted to adjust for possible diurnal variations in the serum levels of hormones (Albertsson-Wikland *et al.*, 1997). The ethics committee of the Faculty of Medicine of the Catholic University of Louvain approved the protocol. The written informed consent of parents and the assent of the participants were obtained.

### Swimming pools and levels of chlorination products

Students of Louvain-la-Neuve had access to a non-chlorinated pool disinfected by copper–silver ionization while students of the two other schools could only visit chlorinated pools. According to the Belgian legislation, each swimming pool is required to regularly check the microbial and chemical quality of water by measuring several parameters including active chlorine (0.5–1.5 ppm) and combined chlorine (<2 ppm). There were some occasional exceedances of active or combined chlorine but without attaining levels that might pose a risk to swimmers according to international guidelines (concentrations of active or combined chlorine never exceeded 4 ppm). Levels of chloroform are not regulated in Belgium. A survey conducted in 2002 on a random sample of 25 public indoor swimming pools in the studied areas reported a median chloroform concentration of 49 μg/L (range, 15–138) in the water and of 33 μg/m^3^ (range, 5–582) in the air of the pools (Charlier *et al.*, 2003). The water of the copper–silver pool was sanitized with concentrations of copper (0.6–1.2 mg/L) and silver (2–10 μg/L) that are in compliance with drinking water guidelines (World Health Organization, 1996).

### Hormonal and protein assays

Total testosterone, sex hormone-binding globulin (SHBG), luteinizing hormone (LH), follicle stimulating hormone (FSH) and dehydroepiandrosterone sulphate (DHEAS) were measured by the Immulite assay (Siemens Medical Solutions Diagnostics, Germany). The free testosterone concentration was estimated by applying the Ly & Handelsman (2005) equations. Inhibin B was measured using the solid-phase sandwich ELISA kit from Oxford Bio-Innovation (OBI), Oxfordshire, UK. To confirm the observations made with this test, we re-analysed B using also the DSL-10-84100i active inhibin B ELISA kit from Diagnostic Systems Laboratories, Inc., Webster, TX, USA. The Pearson correlation coefficient between the inhibin B concentrations (cubic transformed values) measured by the OBI and the DSL methods amounted to 0.63 (*p* < 0.001, regression coefficient, 0.60, *n* = 361). Such a correlation coefficient clearly indicates some noticeable differences between the two assays. We decided thus to use the results obtained with both methods.

### Statistical analyses

Continuous variables are reported as median with interquartile range and normalized by cubic (inhibin B, total and free testosterone) or by logarithmic transformation [body mass index (BMI), LH, FSH, SHGB and DHEAS). Differences in biological parameters between the schools were assessed by one-way anova followed by the Tukey Kramer test. The Kruskal–Wallis non-parametric anova test was used to compare cumulative swimming pool attendance. Binary variables were compared by the chi-square test or by a chi-square test for trend for the analysis of exposure-response relationships. Comparison of inhibin B measured by the OBI or DSL assays was based on least square regression, calculating the Pearson's correlation coefficient. We used stepwise regression analyses to identify factors associated with serum levels of hormones. Regression models were run by testing a broad range of potential predictors, including age, body mass index (BMI, kg/m^2^), time of blood sampling (time of blood sampling in hours), school participation rate, parental educational level defined as father and/or mother graduated from university or a high school (a proxy of socio-economic status), birth weight, maternal smoking during pregnancy, breastfeeding, baby bottle prepared with tap or bottled water, drinking of tap or bottled water, active smoking, use of insecticides at home, distance of home to the nearest busy road and regular practice of a sport other than swimming (e.g. football, basketball, bicycling, horse riding, skiing and tennis). When age and time of sampling did not emerge as significant predictors at the *p*-value of 0.05, they were nevertheless forced in the models on the basis of a priori knowledge. In these analyses, cumulative pool attendance (CPA, hours) was stratified in four categories of lifetime CPA (never, CPA > 0–250, CPA > 250–500 and CPA > 500) and three categories for CPA before the age of 10 years (never, CPA > 0–250 and CPA > 250) or of 7 years (never, CPA > 0–125 and CPA > 125). Three categories were also used to stratify the CPA after the age of 10 years to assess the effects of most recent exposure to swimming pools (never, CPA > 0–250 and CPA > 250). These categories were constructed separately for the attendance of indoor chlorinated pools, outdoor chlorinated pools and of the indoor copper–silver pool of Louvain-la-Neuve. They were tested as dummy variables using as referents the adolescents in the lowest CPA tertile or those having never swum in the swimming pool. For associations identified by multiple regression analyses, we used backward logistic regression models to calculate the odds ratios (ORs) of abnormally low (<10th percentile of values in referents) serum concentrations of inhibin B and total testosterone associated with CPA categories or tertiles before the age of 10 or 7 years. Backward selection started with a model including the same independent variables as in multiple regression analyses and each step was executed by deleting the least significant predictor until the model only contained variables with a *p* < 0.20. *p* values were two-sided and results were considered as statistically significant at *p* values below 0.05. The statistical packages sas 9.1.3 and StatView 5 (Release 5.0.1, 3rd edn., a business unit of SAS, 2001), SAS Institute Inc., Cary, NC, USA were used for all analyses.

## Results

Characteristics of participants are shown in Table 1. Mean age (15.5 years), body weight and birth weight were very similar between the three schools. Compared with their peers of Louvain-la-Neuve, students of Lessines were smaller while those of Bastogne had a higher BMI. The socio-economic status was clearly higher at Louvain-la-Neuve than in the two other schools, as reflected by several lifestyle indicators such as parental education, breastfeeding, exposure to tobacco smoke, use of backyard pools and some sports activities such as skiing or tennis. Because they had access to a copper–silver pool, students of Louvain-la-Neuve had spent much less time in indoor chlorinated swimming pools than their colleagues, 35% of them having even never visited an indoor chlorinated pool. There was, however, no difference between the three schools in the total swimming pool attendance as well as in the proportion of students who were member of a swim club.

**Table 1:** Characteristics of adolescents

	Louvain-la-Neuve, *N* = 162	Bastogne, *N* = 129	Lessines, *N* = 70	*p*
Age, mean (SD), years	15.5 (0.76)	15.5 (0.76)	15.5 (0.85)	0.99
Body weight, mean (SD), kg	63.2 (9.7)	64.5 (11.2)	62.5 (11.3)	0.39
Height, mean (SD), cm	175 (7)	173 (8)	172 (8)*	0.006
Body mass index, kg/m^2^				
Mean (SD)	20.2 (2.4)	21.1 (3.3)*	20.8 (3.0)	0.02
BMI > 25	8 (4.94)	20 (15.5)	5 (7.14)	0.007
Birth weight, mean (SD), kg	3.41 (0.55)	3.33 (0.62)	3.39 (0.60)	0.76
Parental education, *N* (%)	156 (96.3)	87 (68.0)	27 (38.6)	<0.001
Breastfeeding, *N* (%)	136 (84.0)	73 (56.6)	38 (54.3)	<0.001
Parental smoking, *N* (%)	44 (27.0)	50 (39.1)	32 (45.7)	0.01
Maternal smoking during pregnancy, *N* (%)	13 (8.0)	18 (14.1)	11 (15.7)	0.14
Active smoking, *N* (%)	11 (6.8)	9 (7.0)	12 (17.1)	0.03
Ever attendance at outdoor chlorinated pool				
*N* (%)	41 (74.8)	54 (57.8)	30 (42.9)	<0.001
Lifetime CPA (hours, median, IQR)	126 (1.2–392)	23.6 (0.0–168)	0.0 (0.0–347)	<0.001
Ever attendance at indoor copper–silver pool				
*N* (%)	159 (97.5)	1 (0.8)	0 (0.0)	<0.001
Lifetime CPA (hours, median, IQR)	220 (87–426)	31.5	–	<0.001
Ever attendance at indoor chlorinated pool				
*N* (%)	106 (65.0)	128 (100.0)	70 (100.0)	<0.001
Lifetime CPA (hours, median, IQR)	235 (48–640)	536 (309–840)	564 (228–1066)	<0.001
Ever attendance at swimming pool (all types)				
*N* (%)	163 (100.0)	128 (99.3)	70 (100.0)	0.41
Lifetime CPA (hours, median, IQR)	573 (289–989)	536 (309–840)	333 (187–795)	0.75
Other sports *N* (%)				
Basket	3 (1.85)	10 (7.8)	3 (4.29)	0.052
Bicycling	2 (1.24)	8 (6.20)	0 (0)	0.10
Horse riding	2 (1.24)	2 (1.55)	3 (4.29)	0.28
Football	25 (16.0)	43 (33.6)	15 (21.4)	0.001
Tennis	28 (17.3)	3 (2.33)	0 (0.0)	<0.001
Skiing	35 (21.6)	23 (18.5)	8 (11.4)	<0.001

SD, standard deviation; IQR, interquartile range; CPA, cumulative pool attendance.

Practice of other sports was defined as sport club membership except for skiing.

*p* values indicate the levels of statistical significance by the chi-square (prevalences), the Kruskal–Wallis test (CPA) or one-way anova (age and body mass index).

* Significantly different from Louvain-la-Neuve.

Although students of the three schools had virtually the same mean age, levels of serum inhibin B were strikingly different among the three schools. As shown in Table 2, serum inhibin B measured by the OBI or the DSL method was systematically higher at Louvain-la-Neuve than in the two other schools, the difference amounting up to 37% when comparing the DSL inhibin B between Louvain-la-Neuve and Bastogne. The serum levels of total and free testosterone and of LH showed also some variations between the schools but not in the same direction as inhibin B. The concentrations of FSH, SHBG and DHEA were by contrast very similar between the three schools. Blood sampling time was on average the same for Louvain-la-Neuve and Bastogne but it was approximately 1 h earlier at Lessines.

**Table 2:** Concentrations of testicular hormones in the serum of adolescents

	Louvain-la-Neuve (*n* = 163)	Bastogne (*n* = 128)	Lessines (*n* = 70)	*p*
Inhibin B (OBI) (ng/L)	270 (199–339)	225 (171–282)*	222 (177–290)*	0.0002
Inhibin B (DSL) (ng/L)	187 (125–250)	136 (103–185)*	161 (112–232)*,§	<0.0001
Total testosterone (nm)	12.3 (9.4–16.4)	11.3 (7.8–14.6)	13.4 (10.9–17.1)§	0.01
Free testosterone (pm)	192 (116–256)	168 (89.4–239)	208 (141–280)§	0.009
FSH (IU/L)	3.3 (2.3–5.2)	3.6 (2.4–4.8)	4.2 (2.8–5.8)	0.11
LH (IU/L)	1.7 (1.2–2.5)	1.7 (1.1–2.6)	2.1 (1.4–3.1)§	0.03
SHBG (nm)	33.3 (25.1–42.4)	31.8 (25.5–42.5)	31.6 (24.3–44.7)	0.69
DHEAS (mg/L)	1.63 (1.11–2.35)	1.63 (1.22–2.32)	1.75 (1.16–2.27)	0.95
Sampling time (h)	11.3 (9.9–12.5)	11.0 (9.9–14.0)	10.5 (9.5–11.7)	0.02

FSH, follicle stimulating hormone; LH, luteinizing hormone; SHBG, steroid hormone binding globulin; DHEAS, dehydroepiandrosterone sulphate.

The *p* values indicate the level of statistical significance by one-way anova.

* Significantly different from Louvain-la-Neuve.

§ Significantly different from Bastogne.

In stepwise multiple regression analyses, the cumulative attendance of indoor chlorinated pools emerged as the most consistent predictor of serum inhibin B. Whether measured by the OBI or DSL method and adjusted or not adjusted for FSH, the concentration of inhibin B showed a very significant inverse relationship with the time spent in indoor chlorinated pool (Table 3). These associations were already statistically significant with the time spent before the age of 10 and even of 7 years. The only other predictor consistently associated with inhibin B was breastfeeding, which correlated positively with inhibin B, measured by both assays and adjusted or not for LH. Of note, the practice of football or bicycling correlated negatively with the DSL inhibin B, adjusted or not for LH but these sports had no influence on the OBI inhibin B. As shown in Table 4, the concentrations of total and free testosterone decreased with the time spent in indoor chlorinated pools although the associations were not as strong as those emerging with inhibin B. The concentrations of total and free testosterone increased with age and decreased with day time. Interestingly, the practice of horse riding, basket or bicycling was associated with lower levels of total testosterone, adjusted or not for LH. Such associations were also seen with free testosterone except that the influence of bicycling was no more statistically significant after adjustment for LH. Contrarily to inhibin B, total and free testosterone were not associated with breastfeeding.

**Table 3:** Partial regression coefficients (95% confidence interval) between serum inhibin B, before and after adjustment for serum follicle stimulating hormone (FSH), and the attendance of indoor chlorinated pool cumulated over lifetime or before the age of 10 or 7 years

	Inhibin B measured by the OBI method (cubic root)				Inhibin B measured by the DSL method (cubic root)			
		
Cumulative indoor pool attendance (CPA, hours)	Unadjusted^a^		Adjusted^a^		Unadjusted^b^		Adjusted^b^	
	Coefficient (95% CI)	*p*	Coefficient (95% CI)	*p*	Coefficient (95% CI)	*p*	Coefficient (95% CI)	*p*
Lifetime								
>0–250	−0.14 (−0.41 to 0.13)	0.30	−0.15 (−0.39 to 0.10)	0.25	−0.11 (−0.39 to 0.16)	0.42	−0.12 (−0.37 to 0.12)	0.33
>250–500	−0.31 (−0.61 to −0.02)	0.04	−0.33 (−0.60 to −0.06)	0.02	−0.19 (−0.49 to 0.11)	0.22	−0.21 (−0.48 to 0.057)	0.12
>500	−0.35 (−0.64 to −0.05)	0.02	−0.38 (−0.65 to −0.11)	0.006	−0.25 (−0.55 to 0.06)	0.12	−0.28 (−0.56 to −0.010)	0.042
Before the age of 10 years								
>0–250	−0.20 (−0.45 to 0.055)	0.13	−0.21 (−0.44 to 0.01)	0.07	−0.18 (−0.44 to 0.08)	0.16	−0.21 (−0.43 to 0.023)	0.08
>250	−0.23 (−0.37 to −0.09)	0.001	−0.23 (−0.36 to −0.11)	<0.001	−0.19 (−0.33 to −0.05)	0.01	−0.20 (−0.32 to −0.07)	0.002
Before the age of 7 years								
>0–125	−0.22 (−0.45 to 0.009)	0.06	−0.22 (−0.43 to −0.01)	0.04	−0.19 (−0.43 to 0.05)	0.12	−0.19 (−0.40 to −0.02)	0.08
>125	−0.43 (−0.70 to −0.16)	0.002	−0.44 (−0.69 to −0.20)	<0.001	−0.31 (−0.59 to −0.04)	0.03	−0.33 (−0.57 to −0.08)	0.01

All values adjusted for age and time of sampling.

Letters indicate predictors other than indoor pool attendance entering in the model at *p* < 0.05.

a Breastfeeding OR.

b Breastfeeding, football, bicycling. Associations were positive for breastfeeding and negative with bicycling and football.

**Table 4:** Partial regression coefficients (95% confidence interval) between serum total and free testosterone, before and after adjustment for serum luteinizing hormone (LH), and the attendance of indoor chlorinated pool cumulated over lifetime or before the age of 10 or 7 years

	Total testosterone (cubic root)				Free testosterone (cubic root)			
		
	Unadjusted^a^		Adjusted^a^		Unadjusted^a^		Adjusted^b^	
				
Cumulative indoor pool attendance (CPA, hours)	Coefficient (95% CI)	*p*	Coefficient (95% CI)	*p*	Coefficient (95% CI)	*p*	Coefficient (95% CI)	*p*
Lifetime								
>0–250	−0.072 (−0.19 to 0.045)	0.13	−0.076 (−0.18 to −0.024)	0.14	−0.22 (−0.60 to 0.13)	0.26	−0.23 (−0.57 to 0.10)	0.18
>250–500	−0.086 (−0.721to 0.041)	0.19	−0.070 (−0.18 to 0.040)	0.21	−0.38 (−0.80 to 0.035)	0.07	−0.33 (−0.70 to 0.031)	0.047
>500	−0.095 (−0.22 to 0.034)	0.15	−0.12 (−0.23 to −0.007)	0.04	−0.23 (−0.65 to 0.20)	0.29	−0.29 (−0.66 to 0.082)	0.21
Before the age of 10 years								
>0–250	−0.064 (−0.17 to −0.044)	0.25	−0.070 (−0.16 to 0.023)	0.14	−0.20 (−0.56 to 0.16)	0.27	−0.22 (−0.53 to 0.095)	0.17
>250	−0.060 (−0.12 to −0.001)	0.048	−0.063 (−0.12 to −0.01)	0.02	−0.13 (−0.33 to 0.070)	0.21	−0.13 (−0.31 to 0.039)	0.13
Before the age of 7 years								
>0–125	−0.08 (−0.18 to 0.02)	0.11	−0.087 (−0.17 to −0.0003)	0.049	−0.27 (−0.60 to −0.058)	0.11	−0.32 (−0.68 to −0.007)	0.049
>125	−0.130 (−0.25 to −0.014)	0.03	−0.14 (−0.24 to −0.042)	0.005	−0.30 (−0.68 to 0.086)	0.13	−0.29 (−0.58 to −0.001)	0.055

Letters indicate predictors other than indoor pool attendance entering in the model at *p* < 0.05.

a Age, time of blood sampling, horse riding, basket and bicycling.

b Age, time of blood sampling, horse riding and basket. Associations were positive with age and negative with other significant predictors.

The parental education level, a surrogate of the socio-economic status, and the active or passive exposure to tobacco smoke had no influence on serum inhibin B or testosterone. Associations with indoor chlorinated pools were unaltered by the exclusion of subjects who were members of a swimming club or of another competitive sport club (football, tennis and horse riding) (results not shown). Such associations were not found with LH, FSH and other hormones. There were also no significant associations between testicular hormone levels and cumulative attendance of outdoor pools or of the copper–silver pool cumulated during prepuberty or over lifetime time. We also found no significant associations (all *p*> 0.1) between testicular hormones levels and the cumulated attendance of indoor chlorinated pools after the age of 10 years.

**Figure 1:**
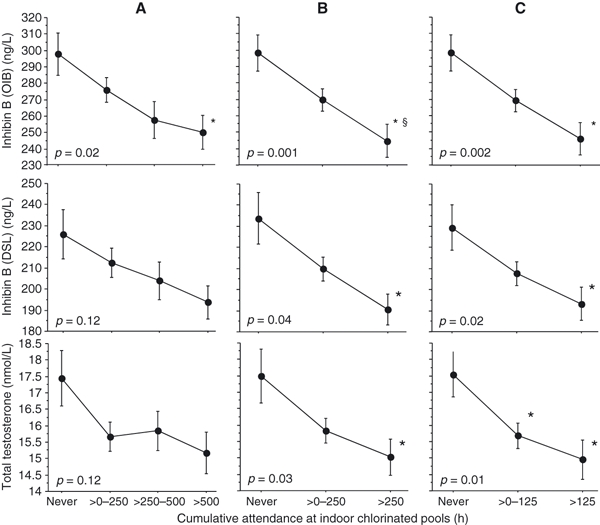
Concentrations of inhibin B and testosterone in the serum of adolescents according to their attendance of indoor chlorinated pools cumulated over lifetime (A), before the age of 10 years (B) or before the age of 7 years (C). Concentrations were adjusted for the following covariates: inhibin B (OBI) for serum follicle stimulating hormone (FSH), age, time of blood sampling and breastfeeding; inhibin B (DSL) for serum FSH, age, day time, breastfeeding, football and bicycling; testosterone for serum luteinizing hormone (LH), age, day time, horse riding, basket and bicycling. Mean ± SE. *Significantly different from the group that never attended an indoor chlorinated pool. §Significantly different from the group that attended an indoor chlorinated pool during >0–250 h.

Figure 1 illustrates the relationships between indoor pool attendance over increasing younger age and the serum levels of inhibin B and testosterone adjusted for gonadotropins and covariates. The mean levels of these two hormones decreased dose-dependently with time spent in indoor pools, especially when the latter was cumulated during prepuberty. The decrease was statistically significant after a CPA > 250 h before the age of 10 years or a CPA > 125 h before the age of 7 years, which corresponds approximately to more than one swimming session of 30 min fortnightly during childhood.

To assess whether these decreases in testicular hormones associated with indoor pool attendance are likely to impact reproductive health, we calculated the odds of having an abnormally low value of serum inhibin B and of total testosterone, or of either hormone according to the time spent in indoor chlorinated pool during childhood. Given the evidence from previous studies that a population of healthy men with unknown fertility includes approximately 10% of subfertile men with serum levels of inhibin B (measured by the OBI test) out of the range found in men with proven fertility (Andersson *et al.*, 2004) we set by analogy the cutoff for low values at the 10th percentile of values observed in our population. We performed this analysis with total testosterone and with the OBI inhibin B, the two hormones most strongly associated with indoor chlorinated pool attendance. Table 5 shows that the odds for low inhibin B were significantly increased in subjects in the highest CPA tertile before the age of 10 years and in those having spent more than 125 h in indoor chlorinated pools before the age of 7 years. Odds for low testosterone were also significantly increased in these two categories as well as in the highest CPA tertile before the age of 7 years. When combining the two hormones, adolescents in the highest CPA tertile or category before the age of 7 or 10 years were two to three more likely to have an abnormally low value of either type of hormone than those in the lowest CPA tertile or those who never swam in indoor chlorinated pools before the age of 7 or 10 years. These analyses brought to light several other predictors (*p* < 0.05), which correlated negatively with testicular hormones: BMI and horse riding for inhibin B, time of blood sampling and basket for total testosterone and BMI, time of blood sampling and basket for the risks of having low value of inhibin B and/or total testosterone. As shown in Table 5, in most cases, there was no association between low values of inhibin B and of total testosterone as a total of 66 subjects had a value of either hormone below the 10th percentile (total should be 36 if decreases of the two hormones were always associated).

**Table 5:** Risks [OR (95% confidence interval)] of low values of inhibin B or total testosterone in serum of adolescents stratified in categories of increasing cumulative attendance of indoor chlorinated pools before the age of 7 or of 10 years

Cumulative pool attendance (hours)			Low inhibin B (OBI) in serum (<133 ng/L)			Low total testosterone in serum (<4.3 nm)			Low inhibin B (OBI) and/or total testosterone in serum		
			
	*N*	Median	*N* (%)	OR (95% CI)^a^	*p*	*N* (%)	OR (95% CI)^b^	*p*	*N* (%)	OR (95% CI)^c^	*p*
Before the age of 7 years											
Never	73	0	4 (5.6)	1		4 (5.5)	1		8 (11.0)	1	
>0–125	199	40	19 (9.6)	1.82 (0.56–5.90)	0.32	17 (8.5)	2.00 (0.55–7.31)	0.3	33 (16.6)	1.42 (0.59–3.44)	0.44
>125	89	211	13 (14.6)	3.49 (1.00–12.1)	0.049	15 (16.9)	5.20 (1.33–20.4)	0.02	25 (28.1)	3.67 (1.45–9.34)	0.006
*p* for trend			0.052			0.01			0.004		
Tertile 1 (0–18)	120	0	7 (5.8)	1		8 (6.7)	1		15 (12.5)	1	
Tertile 2 (19–89)	120	42	12 (10.0)	2.03 (0.72–5.73)	0.18	11 (9.2)	1.72 (0.55–5.39)	0.35	22 (16.7)	1.49 (0.68–3.26)	0.32
Tertile 3 (90–2700)	121	170	17 (14.0)	2.54 (0.94–6.91)	0.07	17 (14.0)	2.79 (0.95–8.22)	0.06	29 (25.6)	2.29 (1.08–4.86)	0.03
*p* for trend			0.03			0.055			0.008		
Before the age of 10 years											
Never	60	0	3 (5.0)	1		4 (6.7)	1		7 (10.6)	1	
>0–250	200	91	19 (9.5)	2.42 (0.65–9.03)	0.19	16 (8.0)	1.50 (0.30–5.72)	0.55	32 (16.0)	1.47 (0.58–3.75)	0.42
>250	101	421	14 (13.9)	3.33 (0.84–13.3)	0.09	16 (15.8)	3.39 (0.85–14.5)	0.08	27 (26.7)	2.83 (1.06–7.52)	0.04
*p* for trend			0.07			0.03			0.01		
Tertile 1 (0–49)	120	2	8 (6.7)	1		9 (7.5)	1		17 (14.2)	1	
Tertile 2 (50–194)	120	126	10 (8.3)	1.38 (0.49–3.85)	0.55	9 (7.5)	1.42 (0.45–4.51)	0.56	17 (14.2)	1.08 (0.49–2.42)	0.84
Tertile 3 (198–4722)	121	364	18 (14.9)	2.54 (0.98–6.62)	0.056	18 (14.2)	2.97 (1.03–8.56)	0.044	32 (26.4)	2.49 (1.19–5.22)	0.02
*p* for trend			0.03			0.056			0.01		

Cut-off for low values of serum inhibin B and total testosterone corresponded to the 10th percentiles of values on the whole population.

a Adjusted for serum FSH*, BMI*, horse riding*, bicycling and age.

b Adjusted for serum LH*, time of blood sampling*, basket*, BMI, bicycling, horse riding, football and tennis.

c Adjusted for serum FSH* and LH*, BMI*, time of blood sampling*, basket*, bicycling, horse riding, football and tennis.

The asterisk indicates predictors entering in the four models of chlorinated pool attendance with a *p* < 0.05.

Although associations found in the analyses mentioned earlier had been adjusted for covariates including parental education, we further ascertained that our results were not confounded by differences in socioeconomic status and quality of life between the schools. For this, we repeated the logistic regression analyses on more homogeneous subpopulations created on the basis of different health and living standard indicators. As shown in Table 6, associations between testicular hormones and indoor chlorinated pool attendance before the age of 7 years persisted across categories obtained by removing adolescents from parents with low education level, adolescents who had been exposed actively or passively to tobacco smoke, adolescents who had not the possibility of holidays skiing (an indicator of higher living standard for Belgium) as well as adolescents who had a BMI > 25 kg/m^2^. Interestingly, exclusion of adolescents with passive or active exposure to tobacco smoke considerably strengthened the associations between testicular hormones and indoor pool attendance. The risk of having low inhibin B and/or total testosterone was indeed almost 10 times higher in subjects with the highest indoor chlorinated pool attendance before the age of 7 years as compared with those who never visited this type of pool during childhood. Risks of low testosterone level were even increased (although not significantly because of the low numbers) with indoor chlorinated pool attendance, when restricting the analysis to the Louvain-la-Neuve school, despite the fact that, in this school selected to recruit a reference population, very few students had regularly attended an indoor chlorinated pool during their childhood (only 15 for more than 125 h before 7 years of age).

**Table 6:** Risks [OR (95% confidence interval)] of low values of inhibin B or total testosterone in serum of adolescents stratified in categories of increasing cumulative attendance of indoor chlorinated pools before the age of 7 years

	Cumulative pool attendance (hours) before the age of 7 years								
		
	Never		>0–125			>125			
				
	*N* (%)	OR	*N* (%)	OR (95% CI)	*p*	*N* (%)	OR (95% CI)	*p*	*p* for trend
Low inhibin B (<133 ng/L)									
High parental education	4/68 (5.88)	1	15/144 (10.4)	2.06 (0.59–7.21)	0.26	8/58 (29.6)	2.95 (0.74–11.8)	0.13	0.14
No passive or active smoking	2/48 (4.17)	1	10/106 (8.62)	2.58 (0.46–14.6)	0.28	7/46 (15.2)	7.06 (1.11–44.8)	0.04	0.07
No skiing	2/42 (4.76)	1	11/126 (8.73)	1.66 (0.34–8.13)	0.53	12/60 (20.0)	4.24 (0.86–21.0)	0.08	0.01
BMI < 25 kg/m^2^	4/69 (5.80)	1	14/179 (7.82)	1.48 (0.44–4.95)	0.52	10/80 (12.5)	2.86 (080–10.23)	0.11	0.14
Louvain-la-Neuve	4/67 (5.97)	1	6/80 (7.50)	1.22 (0.26–5.83)	0.80	1/15 (6.79)	1.49 (0.12–18.4)	0.75	0.79
Low total testosterone (<4.3 nm)									
High parental education	4/68 (5.88)	1	14/144 (9.72)	1.95 (0.52–7.32)	0.32	10/58 (17.2)	4.38 1.04–18.5)	0.04	0.04
No passive or active smoking	2/48 (4.17)	1	5/116 (4.31)	1.11 (0.17–7.26)	0.92	11/46 (23.9)	7.39 (1.11–49.2)	0.04	<0.001
No skiing	2/42 (4.76)	1	10/126 (7.94)	2.33 (0.31–17.4)	0.17	8/60 (13.3)	9.76 (1.42–67.0)	0.02	0.19
BMI < 25 kg/m^2^	4/69 (11.6)	1	14/179 (7.82)	1.81 (0.48–6.81)	0.38	14/80 (17.5)	4.74 (1.19–18.9)	0.03	0.01
Louvain-la-Neuve	4/67 (5.97)	1	6/80 (7.5)	1.55 (0.27–8.90)	0.63	3/15 (20.0)	7.59 (0.73–78.9)	0.09	0.15
Low inhibin B and/or total testosterone									
High parental education	8/68 (11.8)	1	26/144 (18.1)	1.50 (0.60–3.72)	0.39	17/58 (29.3)	3.41 (1.25–9.25)	0.02	0.01
No passive or active smoking	4/48 (8.33)	1	14/116 (12.1)	1.30 (0.35–4.79)	0.7	17/46 (37.0)	9.14 (2.39–35.0)	0.001	<0.001
No skiing	4/42 (9.52)	1	19/126 (15.1)	1.48 (0.42–5.23)	0.54	17/60 (28.3)	4.21 (1.15–15.5)	0.03	0.01
BMI < 25 kg/m^2^	8/69 (11.6)	1	26/179 (11.9)	1.24 (0.50–3.07)	0.64	21/80 (26.3)	3.19 (1.23–8.30)	0.02	0.01
Louvain-la-Neuve	8/67 (11.9)	1	12/80 (15.0)	1.73 (0.54–5.59)	0.36	4/15 (26.7)	5.17 (0.98–27.3	0.36	0.2

ORs were adjusted for the same predictors as in Table 5.

## Discussion

Serum hormones levels have proved to be validated surrogate markers of testicular function and sperm quality in epidemiological studies in which the collection of semen cannot be performed (Andersson *et al.*, 2004; Jensen *et al.*, 1997). While reflecting reliably spermatogenesis, serum hormones are also not prone to unavoidable selection bias because of the low participation in population-based studies on semen quality. By applying such markers in a cross-sectional study on male adolescents, we unexpectedly found strong inverse associations between the serum levels of inhibin B and total testosterone and the cumulative attendance of indoor chlorinated swimming pools. These associations were most strong with the time spent in indoor pools during childhood and when serum levels of inhibin B and total testosterone were adjusted for gonadotropins. Subjects with the highest pool attendance during childhood had concentrations of inhibin B and testosterone on average 20% lower than those who never attended indoor chlorinated pool. These adolescents were about three times more likely to have abnormally low inhibin B or testosterone levels in serum. Such associations were not seen with the serum levels of LH and FSH nor with SHBG, DHEAS, which suggests that they are caused by changes occurring primarily in the testes.

Our study was based on school populations, which differed not only according to indoor chlorinated pool attendance but several other lifestyle-related factors that might affect reproductive health. The socio-economic status, as evaluated by parental education level and, as reported previously (Richthoff *et al.*, 2008; Ramlau-Hansen *et al.*, 2008), the active or passive exposure to tobacco had no direct influence on the serum levels of inhibin B and testosterone. Several other lifestyle-related predictors of testicular hormones were however identified by our study, among which the most consistent one was breastfeeding, which correlated positively with the values of inhibin B measured by both assays irrespective of the adjustment for FSH. We have no explanation for this positive association, which might reflect a protective effect of breastfeeding or perhaps a detrimental effect linked to bottled milk. In agreement with previous studies (MacDonald *et al.*, 2010), we also found a negative influence of BMI in particular in the risk of low inhibin B values. Interestingly, the practice of some sports such as horse riding, bicycling or basket was also associated with lower levels of inhibin B or of testosterone. Although these associations might be causal and attributed to a mechanical stress on the testes (multiple injuries, compression shorts, etc) they must be interpreted cautiously as they were based on small numbers of subjects.

Swimming or bathing in itself cannot explain such associations, which were not found with the attendance of outdoor chlorinated pools or of the copper–silver pool. Disruption of the male reproductive hormonal profiles by intensity of swimming does not appear either to be the explanation. Differences in testicular hormones between the three schools were observed despite a similar lifetime attendance at swimming pools. Associations with indoor chlorinated swimming pools also persisted after exclusion of subjects training intensively in swim clubs and they were already very significant with pool attendance cumulated till the age of 7 years, that is, when children could not really swim. Last, the pattern of hormonal changes found in our study is distinct from that caused by intense exercise (Safarinejad *et al.*, 2009), which consists in a reversible decrease not only of testosterone and inhibin B but also of LH and FSH.

If decreases in testicular hormones associated with the attendance of chlorinated indoor pools cannot be explained by the intensity of swimming, then they should be caused by a factor specifically linked to indoor chlorinated pools. The only factor that might affect reproductive health and that distinguishes indoor chlorinated pools from the other types of pool is the amount of CBPs released during the chlorination of organic matter brought by swimmers. Because of their higher bather load, public indoor pools are indeed much more heavily polluted by CBPs than residential pools and *a fortiori* than pools disinfected by the copper–silver method. Public pool water contains a variety of potential reprotoxicants among which the HANs, HAAs and THMs are the most abundant. These CBPs might quite conceivably damage the testes, especially the Leydig and Sertoli cells, which have long been known to be sensitive to toxic insults (Steinberger & Klinefelter, 1993). Another potential target of CBPs and in particular of chloramines is the blood–testes barrier (Mruk & Cheng, 2010; Lie *et al.*, 2009), a structure formed by the tight junctions linking Sertoli cells, which is also a frequent target in chemical-induced testicular injury (Zhang *et al.*, 2008; Siu *et al.*, 2009). Chloramines are indeed membrane penetrating oxidants that can rapidly open tight junctions (Musch *et al.*, 2006; Bernard, 2007). At the high concentrations found in public pool water, these CBPs might conceivably disrupt the blood–testes barrier and thereby decrease the viability and number of Sertoli cells. If one assumes that CBPs can cause damage to the blood-testes barrier or to the Leydig or Sertoli cells, these effects must necessarily vary with the concentrations of these reprotoxicants in pool water and hence with the level of organic pollution and the type of swimming pool. These effects also depend on the uptake and metabolism of these chemicals by the testes while the resulting changes in the serum levels of inhibin B and testosterone levels probably vary with feedback mechanisms controlling the production of these hormones. All these sources of individual variation might explain why decreases in inhibin B and total testosterone occur independently in the majority of subjects.

Extrapolation from animal studies suggests that CBPs are unlikely to be detrimental to the testes of swimmers at the doses that can be taken up in a swimming pool. If one refers to the risk assessment performed for chloroform, the highest dose of CBPs a child can absorb during a swimming session is more than two orders of magnitude lower the doses causing testicular damage in rodents (between 10 and 100 mg/kg^2^) (Smith *et al.*, 1986; Linder *et al.*, 1994, 1995, 1997; Graves *et al.*, 2001; Christian *et al.*, 2002; Abdel-Nahab, 2003). This assessment, however, is based on the systemic uptake of CBPs and does not make account of the direct uptake of CBPs across the scrotal skin nor on the sensitivity of the testes which might be higher in man than in rodents. When one considers the concentration of CBPs in swimming pool water, the lipophilic properties of these chemicals and the very high permeability of the scrotum compared with the rest of body, the direct uptake of CBPs by the testes could quite possibly lead to tissue concentrations in the same range as those causing testicular damage in experimental animals exposed by systemic route. The hypothesis of a transdermal testicular toxicity appears especially plausible as swimming pool water offers ideal conditions to skin absorption. The high temperature of the water (up to 35 °C in the small pool), the hydratation of the skin and the disrupting effects of chlorine on the skin barrier (Seki *et al.*, 2003) are indeed all factors that in combination should greatly facilitate the permeation of CBPs across the scrotum. In that respect, it is interesting to note that human exposure to CBPs peaks during childhood when children cannot swim and have thus to attend the small pool, which is warm and heavily polluted by CBPs. This means that the exposure of the testes to CBPs culminates during the prepubertal period, precisely when the male reproductive system is under development and most vulnerable to toxic insult. This unfortunate coincidence between exposure to CBPs and sensitivity of the testis to hormone deregulation might explain why serum levels of inhibin B and testosterone are most strongly associated with pool attendance cumulated before the age of 10 or 7 years.

The main limitation of our study concerns the exposure assessment even though we based our analysis on the number of hours adolescents had spent in the different types of swimming pools. It was of course impossible to retrieve exposure data regarding the actual levels of CBPs in swimming pools that were attended our adolescents. This is especially true as, at the exception of chloramines, CBPs are not regulated in Belgian swimming pools. The finding of strong and consistent associations between testicular hormones, especially inhibin B, and the attendance of indoor chlorinated pool suggests however that the lack of data about levels of CBPs in pool water has not been critical to the point of distorting exposure–response relationships. Another possible limitation in this population based study conducted in schools is that for ethical reasons, it was of course not possible to obtain information about the Tanner stage or about the testes size. The fact that these tests were not performed is more an advantage than an inconvenience. Such tests applied in schools would have certainly deterred a large fraction of the parents and adolescents to participate in the study. But in addition, our participants were blinded to the tested hypothesis, which allowed us to minimize the participation and response biases, which indeed are important in that type of investigation.

Inhibin B is a reliable index of Sertoli cell population correlating with the testes volume and the sperm count (Meachem *et al.*, 2001; Kumanov *et al.*, 2006). The number of Sertoli cell and thus the levels of inhibin B are definitively set at puberty when multiplication of Sertoli cells stops and when spermatogenesis starts. As shown by Andersson *et al.* (1997), the adult level of serum inhibin B has been reached by the pubertal stage II (mean age 12.6 years in the study by Andersson *et al.* (1997). The low serum level of inhibin B as observed in adolescents (age 14 to 18) with the highest indoor pool attendance is thus predictive of a reduced capacity of sperm production in adult life (Traish *et al.*, 2009; Diaz-Arjonilla *et al.*, 2009). The risk of decreased fertility concerns especially adolescents with serum inhibin B below the 10th percentile as it has been shown that populations of healthy men with unknown fertility includes approximately 10% of subfertile men with serum levels of inhibin B out of the range found in fertile men (World Health Organization, 1996). By contrast, total testosterone and LH in serum continue to increase to the last stage of puberty [V, mean age of 17 years in the study by Andersson *et al.*, (1997)] meaning that changes reported in our study were observed at a time Leydig cell maturation was still ongoing. Furthermore, damaged Leydig cells have the potential to regenerate, which is not the case of Sertoli cells for which any loss is irreversible. For these reasons, the lower levels of total testosterone in adolescents having attended chlorinated pools during their childhood should be interpreted with caution. This is especially true as the cutoff level used to define these values was, as explained above, set at the 10th percentile of values in our population on the basis of observations made with inhibin B in adults. These lower values might thus merely reflect a delayed in Leydig cell maturation, which is not necessarily predictive of abnormally low levels at adult age. However, one should not infer from these considerations that the decreased levels of testosterone observed in our study have no clinical significance. There is indeed a large amount of data associating low testosterone levels to a variety of adverse outcomes, including outcomes that can develop during childhood and puberty such as retarded growth or prediabetic state (Meachem *et al.*, 2001; Kumanov *et al.*, 2006).

In conclusion, our study found strong inverse relationships between testicular hormones and the attendance of indoor chlorinated pools during childhood, which suggests that this type of pool contains substances detrimental to the testes. Although these findings need to be replicated by independent studies, they stress the importance of enforcing regulations and controls of CBPs in swimming pools, especially in pools attended by young children.
